# Immunoregulation role of the erythroid cells

**DOI:** 10.3389/fimmu.2024.1466669

**Published:** 2024-10-15

**Authors:** Chunxiao Niu, Jiyan Zhang

**Affiliations:** Department of Molecular Immunology, Beijing Institute of Basic Medical Sciences, Beijing, China

**Keywords:** immunoregulation, erythropoiesis, CD71 + erythroid cells, ROS, PD-L1, TGF-β

## Abstract

Erythroid cells are the most abundant cells in the human body. In addition to their established function in gas-transportation, erythroid cells at various stages of differentiation have recently been shown to have immunomodulatory roles. Red blood cells may serve as modulators of innate and adaptive immunity, while their immature counterparts, CD71^+^ erythroid cells (CECs) have important immunomodulatory functions in various contexts. CECs are abundant in human cord blood and placenta, where they contribute to fetomaternal tolerance. CECs also accumulate in patients with infections, tumors, and anemia, and effectively suppress T cells by producing high levels of arginase, reactive oxygen species, programmed death-ligand 1, transforming growth factor β, and/or interleukin-10. Here, we systematically summarize the immunomodulatory functions of erythroid cells and propose some potential therapeutic applications based on their characteristics.

## 1 Introduction

In vertebrates, red blood cells (RBCs) are abundant in the circulation and are the main medium for oxygen transportation in the blood. In recent years, several studies have demonstrated that erythroid cells have additional functions beyond oxygen transport. Given their high level of production, vast numbers, and whole-body distribution, understanding of the immunomodulatory effects of erythroid cells has potential to provide novel targets for future immunotherapy approaches.

The immunoregulatory effects of erythroid cells were first discovered over 70 years ago, in 1953, when Nelson RA Jr discovered the phenomenon of immune-adherence between microorganisms and erythrocytes, which caused an immunologically specific reaction and enhanced phagocytosis (1). Subsequently, in 1979, the immunosuppression mediated by splenic nucleated erythrocytes was first revealed (2), followed by the work of Conway de Macario in 1980, linking immunosuppression with erythropoiesis in irradiated spleen-cell-transferred C57BL/6J mice (3). These studies revealed that nucleated erythrocytes can suppress primary and secondary antibody-mediated responses *in vivo* (4). A few years later, nucleated erythrocytes, which inhibit B-cell proliferation in humoral immune responses, were named erythroid immunosuppressor cells (5). Recent studies have demonstrated that erythroid cells modulate both innate and adaptive immune responses (6). The aims of this review were to introduce the basic features of erythropoiesis and to summarize the immunomodulatory functions of RBCs and CD71^+^ erythroid cells (CECs).

## 2 Erythropoiesis

Erythropoiesis is a constant, multi-stage process, which takes approximately 14 days in adult humans, who produce almost 200 billion RBCs every day, while mice generate more than 7000 erythrocytes per second (7, 8). During adulthood, steady state RBC generation occurs in the bone marrow, while damaged and/or senescent RBCs are recognized, internalized, and digested by splenic red pulp macrophages and Kupffer cells in the liver. This cycle of production and clearance creates steady-state RBC life spans of approximately 120 and 60 days in humans and mice, respectively (9).

### 2.1 Developmental stages of erythropoiesis

#### 2.1.1 Embryonic hematopoiesis

**Figure 1:**
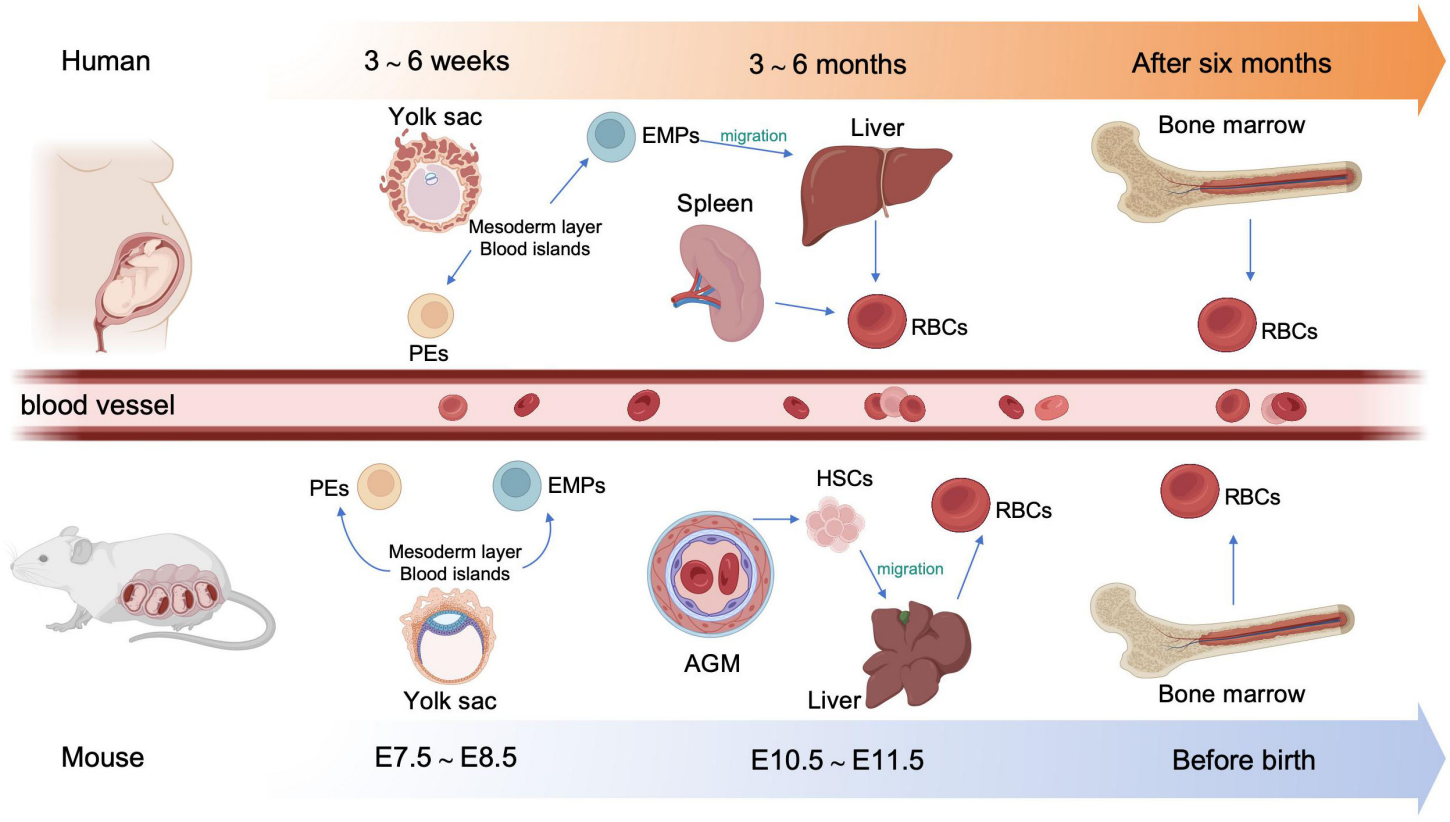
Overview of embryonic hematopoiesis in human and mouse. In human, erythropoiesis first occurs in the blood islands from the mesoderm layer of the yolk sac, generating primitive erythroblasts (PEs). Subsequently, erythromyeloid progenitors (EMPs) from the yolk sac migrate to the fetal liver and spleen. Finally erythropoiesis occurs in bone marrow. In mouse, primary erythropoiesis develops in the yolk sac. The yolk sac then atrophies and hematopoietic stem cells (HSCs) appear in aorta-gonad-mesonephros (AGM) region and transfer to the liver. Finally, erythropoiesis transfers to the bone marrow before birth.

In human embryos, erythropoiesis first occurs in the yolk sac, then transfers to the fetal liver and spleen, and finally becomes established in the bone marrow (10). Blood islands form from the mesoderm layer in the yolk sac, where primitive erythroid progenitor cells differentiate into primitive erythroblasts (PEs), which produce embryonic hemoglobin (α2ϵ2) (11). During weeks 6–8 of gestation, erythro-myeloid progenitors (EMPs) from the yolk sac begin to transfer to the fetal liver and spleen. The liver becomes the primary site of erythropoiesis during weeks 10–28 of gestation, while the spleen is the primary producer of RBCs during the second trimester (12, 13). At the end of the second trimester, erythropoiesis transfers to the bone marrow, which becomes the primary site of erythropoiesis until birth; fetal hemoglobin is produced to facilitate oxygen transport across the placenta during this stage (14). After birth, fetal hemoglobin output gradually decreases and is replaced by the adult form of hemoglobin. (**Figure 1**).

In mouse embryos, hematopoiesis first emerges in the yolk sac at embryonic day 7.5 (E 7.5), and is characterized by the production of PEs, with diploid platelet progenitor cells and macrophages (15). Subsequently, EMPs emerge in the yolk sac at approximately E 8.25, which can generate erythroid colonies similar to those derived from adult bone marrow and have the capacity to produce multiple other myeloid lineages (16, 17). Soon afterwards, at around E10.5, hematopoietic stem cells (HSCs) appear in the dorsal aorta of the aorta-gonad-mesonephros region. Meanwhile, HSCs may also emerge from other hemogenic endothelial cells (ECs) within arteries in the umbilical cord, yolk sac, vitelline, cranial, and placental regions. These HSCs then migrate to the fetal liver, where they undergo a period of expansion, until they transfer to the bone marrow before birth (18) (**Figure 1**).

#### 2.1.2 Stages of erythroid development

The development of erythroid cells during erythropoiesis can be divided into five stages. During the first stage, HSCs differentiate into megakaryocyte-erythroid progenitors (MEPs). The second stage is initiated by the differentiation of erythroid progenitor cells, followed by the appearance of burst-forming unit-erythroid (BFU-E) progenitors, and ends with the differentiation of colony-forming unit-erythroid (CFU-E) progenitors (19). The third stage begins with the development of pro-erythroblasts, followed sequentially by basophilic erythroblasts (Baso-E), polychromatic erythroblasts (Poly-E), and orthochromatic erythrocytes (Ortho-E). The fourth stage comprises reticulocytes, with mature erythroid cells formed in the fifth and final stage. Reticulocytes mature in the bone marrow, where they begin to eliminate mitochondria and other organelles, and subsequently enter the circulation to undergo further maturation into erythrocytes (20, 21). Erythroid cells gradually reduce their overall and nucleus size, while simultaneously increasing their hemoglobin content (10). Markers of erythropoiesis are listed in **Table 1**.

**Table 1:** Cell markers of the erythropoiesis.

Cell type Species		Cell surface markers	Ref.
HSC	Human	Lin^-^CD34^+^CD38^-^CD45RA^-^Thy1^+^CD49f^+^	(22, 23)
ST- HSC	Mouse	c-Kit^+^Lin^-^Sca-1^+^Flk-2^-^Flt3^-^CD34^+^	(24, 25)
LT- HSC	Mouse	c-Kit^+^Lin^-^Sca-1^+^CD150^+^CD48^-^CD41^-^CD34^-^	(24, 25)
MEP	Human	Lin^-^CD34^+^CD38^+^CD10^-^CD45RA^-^CD135^-^	(26)
	Mouse	Lin^-^IL17rα^-^c-Kit^+^Sca-1^-^CD34^-^CD16/32^-^	(27)
BFU-E	Human	CD45^+^GPA^-^IL-3R^-^CD34^+^CD36^-^CD71^low^	(28–30)
	Mouse	CD45^+^CD150^+^c-Kit^+^ Sca-1^-^CD71^low^	(28, 29, 31)
CFU-E	Human	CD45^+^GPA^-^IL-3R^-^CD34^-^CD36^+^CD71^high^	(28, 30)
	Mouse	CD45^-^c-Kit^+^TER119^-^CD71^hi^	(28, 31)
Erythroblasts	Human	CD45^-/+^CD235a^+^CD71^hi^c-Kit ^low/-^	(28, 32)
	Mouse	CD45^-/+^TER119^+^CD71^hi^c-Kit^low/-^CD44^+^	(33, 34)
Retic	Human	CD235a^+^CD71^+^RNA^+^DNA^low^	(35, 36)
	Mouse	TER119^+^CD71^+^RNA^+^DNA^low^	(35, 37)
Erythrocytes	Human	CD45^-^CD235a^+^CD71^-^DNA^low^	(35)
	Mouse	CD45^-^TER119^+^CD71^-^DNA^low^	(35, 37, 38)

### 2.2 Molecular regulation of erythropoiesis

The differentiation of HSCs to erythroid cells is regulated by various cytokines and growth factors (**Table 2**). The first stage of erythropoiesis is regulated by hematopoietic cytokines, such as stem cell factor (SCF; also known as c-Kit ligand), granulocyte-macrophage colony-stimulating factor, interleukin-3 (IL-3), thrombopoietin, IL-11, and transforming growth factor β (TGF-β). Further erythropoiesis is mainly regulated by erythropoietin (EPO), and iron metabolism is essential for hemoglobin synthesis. GATA1, GATA2, KLF1, and TAL1 are key transcription factors involved in erythropoiesis, while the transcription factors, FOG1, and BCL11A, regulate the expression of genes encoding enzymes associated with heme biosynthesis and hemoglobin production (62). Factors involved in the erythropoiesis are listed in **Table 2**.

**Table 2:** Factors involved in the erythropoiesis.

Factor	Effect	Ref.
Transcriptional factors		
GATA1	Regulating the survival and terminal differentiation of EPCs.	(39, 40)
GATA2	Regulating the proliferation and maintenance of HSCs and progenitor cells	(41)
KLF1	Inhibiting the differentiation of megakaryocytes while promoting early differentiation of erythroid cells.	(42)
TAL1	Promoting the differentiation of erythroid cells and contributing to the formation of distinct gene regulatory complexes in EPCs.	(43)
Growth factors		
SCF	Activating downstream signaling proteins PI3K and Akt to influence cellular survival; Indirectly phosphorylating EPO-R to activate the EPO/EPO-R signaling pathway.	(11, 21, 44)
GM-CSF	Inducting the division and differentiation of BFU-Es into CFU-E cells.	(45)
EPO	Regulating late stages of erythropoiesis mainly through EPOR-JAK2-STAT5/STAT3 signaling pathway.	(46–48)
TPO	Affecting the rate of entry into the cell cycle and proliferative capacity of HSCs.	(49)
IL-3	Supporting the proliferation of early progenitors stimulated by autocrine TGF-β.	(50–52)
IL-11	Promoting the growth of early progenitors and increasing platelet production.	(53)
TGF-β	Promoting CD34^+^ HSPCs differentiation into EPCs; Suppressing the proliferation of CECs.	(54–57)
Factors modulating iron metabolism		
Iron	Material for hemoglobin synthesis.	(58, 59)
Ferroportin	Iron exporter.	(60)
Hepcidin	Binding to iron exporter ferroportin to induce its internalization and degradation	(60)
Erythroferrone	Suppressing the synthesis of hepcidin to allow iron mobilization to facilitate erythropoiesis.	(61)

PI3K, phosphoinositide-3 -kinase; Akt, protein kinase B; TGF-β, transforming growth factor-β; HSPCs, hematopoietic stem and progenitor cells; EPCs: erythroid progenitor cells; CECs: CD71^+^ erythroid cells; JAK2: Janus kinase2.

### 2.3 Macrophages in erythropoiesis and erythrophagocytosis

#### 2.3.1 Erythroblast islands

Erythroblastic islands (EBI), first discovered by Marcel Bessis in 1958, provide a specialized microenvironment for erythropoiesis (63). EBIs contain a central macrophage surrounded by maturing erythroblasts, and act as the erythroid precursor niche, which supports the bone marrow in producing RBCs at a rate of 2.5 million/second at homeostasis in adult humans (7). Terminal erythroid differentiation occurs within EBIs, where late CFU-Es mature into reticulocytes (64). Macrophages in the EBI secrete growth factors to support erythropoiesis, provide iron for hemoglobin synthesis, phagocytose extruded nuclei, and prevent toxic effects of free DNA (65, 66). Both mouse and human EBI macrophages express EPO-R, while EPO in the niche acts on erythroid cells and EBI macrophages simultaneously, to promote erythropoiesis. Under stress conditions (see section 2.4), RBCs are mainly produced through splenic erythropoiesis, which is distinct from bone marrow steady-state erythropoiesis (67). Impaired EPO-R signaling in splenic niche macrophages significantly inhibits the differentiation of stress erythroid progenitors (68). Further, EBI macrophage dysfunction can lead to specific erythroid hematological disorders (69).

#### 2.3.2 Erythrophagocytosis

RBCs have a life span of around 120 days in the circulation. Macrophages have important roles in phagocytosis of aged or injured RBCs and contribute to iron recycling (70). RBC clearance is regulated by so called “eat me” and “don’t eat me” signals. Interaction of CD47 with SIRPα provides the “don’t eat me” signal (71). When RBCs undergo aging, “eat me” signals, such as phosphatidylserine (PS) and band 3, accumulate on their membranes in a process termed eryptosis (72). PS binds to Tim-1, Tim-4, Stabilin-2, or CD300 on macrophages, generating a pro-phagocytic signal, while band 3 interacts with CR-1 and Fc receptors to facilitate phagocytosis (73). PS also binds to platelets and ECs, which triggers pro-thrombotic risk and compromises the microcirculation (72). Enhanced eryptosis is observed in several clinical conditions, including malignancies (72). Tumor cells can directly interact with RBCs via galectin-4, leading to RBC aggregation (74). Together, RBC aggregation and augmented RBC adherence to the vascular wall due to enhanced eryptosis enable circulating tumor cells to stably roll along the vessel wall at a lower flow rate (75).

### 2.4 Stress erythropoiesis

**Figure 2:**
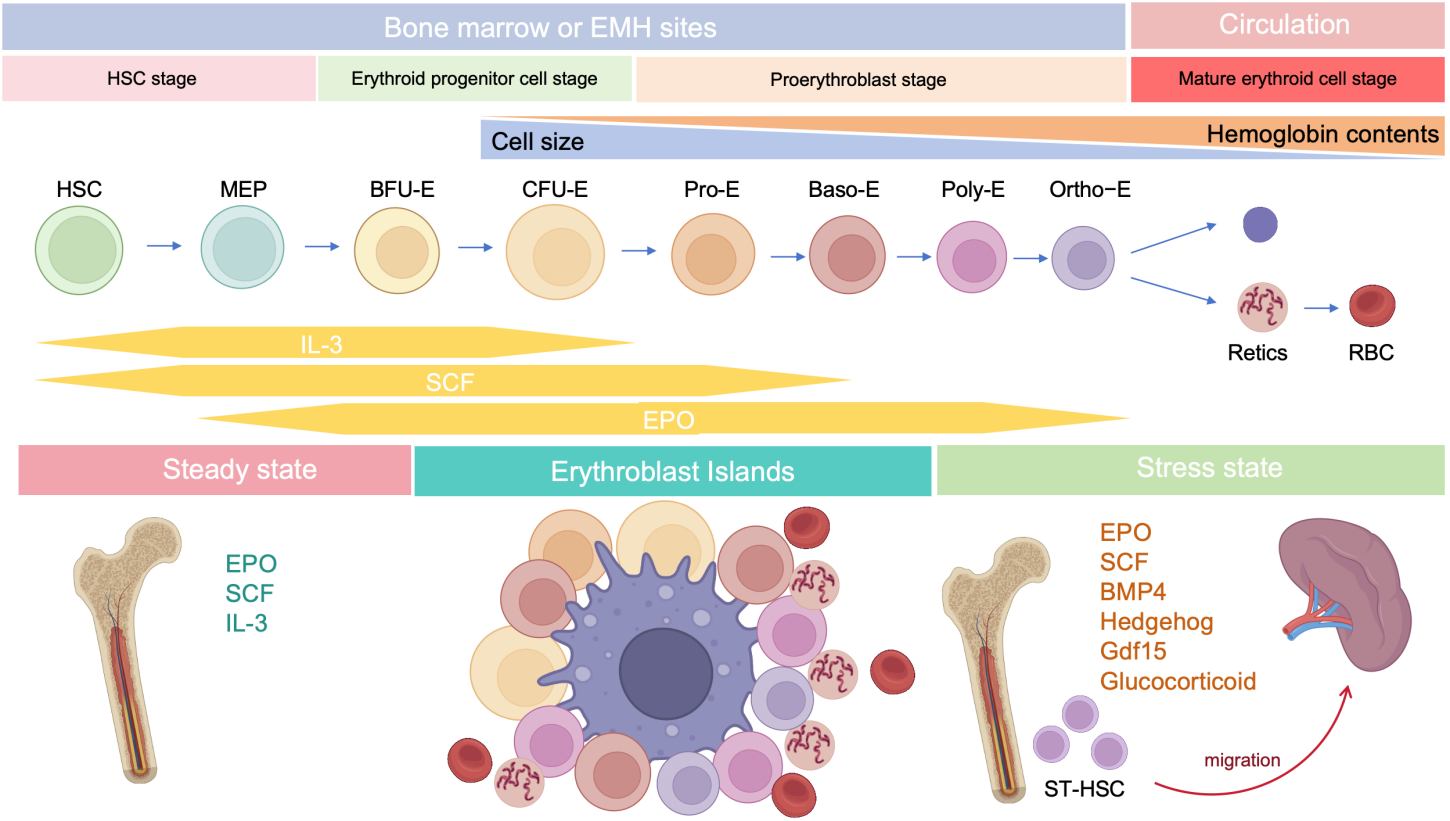
Developmental stages of erythropoiesis after birth. Under steady state, erythropoiesis occurs in the bone marrow, while stress erythropoiesis occurs mainly in the spleen. Erythropoiesis occurs in erythroblastic islands, which contains a central macrophage surrounded by developing erythroid cells.

Stress erythropoiesis is a stem cell-based tissue regeneration response that occurs in the spleen and fetal liver (76). Anemia or hypoxia accompanied by inflammation, which occur frequently during cancer development (77, 78), chronic infection (79), severe trauma (80), and chronic psychological stress (81, 82), disrupt the homeostasis between erythroid cell production through steady-state erythropoiesis and clearance of senescent or damaged erythroid cells by phagocytes, inducing stress erythropoiesis (79, 83); this process is regulated by bone morphogenetic protein 4 (BMP4), SCF, Hedgehog, EPO, growth-differentiation factor 15 (Gdf15), and glucocorticoids (GCs) (**Figure 2**) (84). Under homeostatic conditions, low EPO levels support terminal differentiation of only the most EPO-sensitive progenitors, while other erythroid progenitors undergo apoptosis; however, during stress erythropoiesis, increased EPO levels induce massive and rapid terminal differentiation of all erythroid progenitors (56). In addition, BMP4 and Hedgehog signals restrict the transition of short-term-HSCs to EPO-sensitive stress erythroid progenitors. Immature stress-induced erythroid progenitors maintain stem cell properties, including self-renewal, and can be serially transplanted (84–87). Further, BMP4 and SCF are required for the expansion of stress BFU-E spleen cells under hypoxic conditions (88), while Gdf15 regulates murine stress erythroid progenitor proliferation and controls stress erythropoiesis niche development (89). GCs are also essential for immature erythroid cell expansion during stress erythropoiesis, and act by binding and modulating the transcriptional activity of their cognate nuclear receptor, glucocorticoid receptor (GR) (90).

Although biomarkers of BFU-E erythroid progenitors (Lin^-^cKit^+^CD71^Low^CD150^+^CD9^+^Sca-1^-^) responsive to stress erythropoiesis in the murine spleen are the same as those detected during steady state (91), whole genome transcriptional analysis demonstrated that mouse stress-BFU-E gene signatures are more BMP4-responsive and associated with erythropoiesis and proliferation, relative to those detected in the steady-state (92). Single-cell RNA-seq analysis of human stress-induced erythroid progenitors also revealed a distinct sub-population to that observed under steady-state erythropoiesis (93). Furthermore, splenic BFU-E exhibit different growth properties to their bone marrow counterparts; splenic BFU-E require only EPO to form colonies, while bone marrow BFU-E require EPO and a second factor (94).

Factors upstream of stress erythropoiesis have fundamental immunomodulatory effects. EPO is the principal cytokine regulating erythropoiesis through EPOR; however, EPOR is expressed not only on erythroid cells, but also on immune cells, such as macrophages, dendritic cells (DCs), mast cells, and lymphocytes (40). EPO can bind to EPOR and tissue-protective receptor (TPR, an EPOR/CD131 heterodimer), which are important in tissue protection and immune regulation (95). EPO inhibits the induction of genes encoding proinflammatory factors, such as TNF-α and inducible nitric oxide (NO) synthase (iNOS), in activated macrophages by decreasing NF-κB p65 activation (96). In addition, EPO suppresses DC maturation through the Jak2/STAT-3/SOCS1 pathway (97). Furthermore, EPO directly promotes regulatory T cell (Treg) proliferation, while inhibiting the expansion of conventional T cells via molecular crosstalk with the IL-2 pathway (98). GCs are required for regulation of stress erythroid progenitor expansion (90); however, GR signaling also has potent anti-inflammatory effects (99). Stress erythropoiesis produces more RBCs and CECs, and both populations possess considerable immunomodulatory functions under various conditions (see below for further details).

## 3 Immunomodulatory effects of RBCs

**Figure 3:**
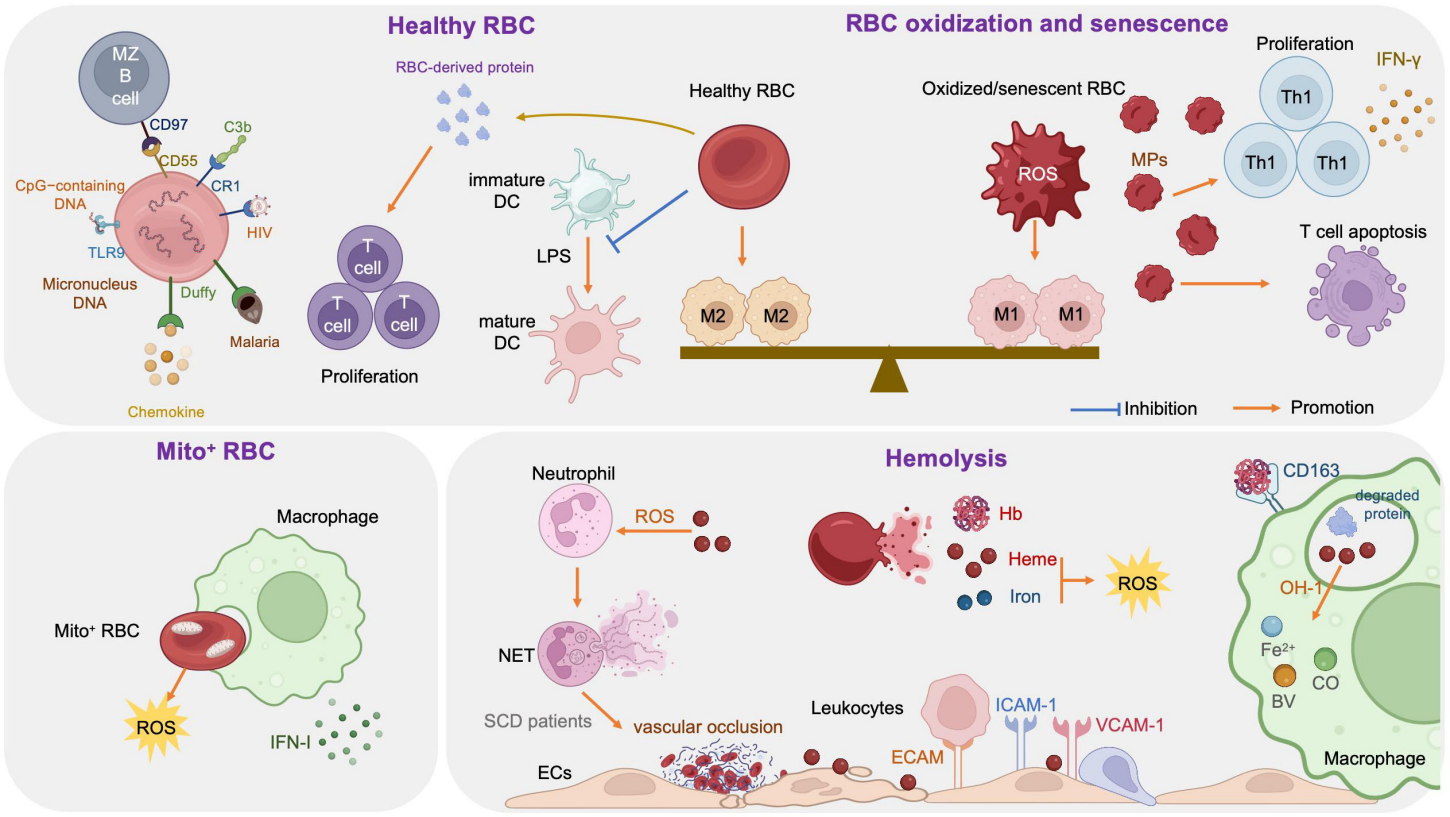
Immune regulation effects of red blood cells (RBCs).

The link between RBCs and immune function was reported as early as 1953, when Nelson RA Jr. discovered the phenomenon of immune-adherence between erythrocytes and microorganisms, which augments phagocytosis (1). In 1991, RBCs were reported to bind to IL-8 and prevent its release into the blood, thereby limiting leukocyte stimulation (100). Data reported in 1993 demonstrated that RBCs can bind to several chemokine superfamily inflammatory peptides, indicating that RBCs may act as regulators of inflammatory processes (101). Unlike healthy RBCs, RBCs carrying mitochondria (Mito^+^ RBCs) augment inflammation. Furthermore, oxidative stress and RBC senescence generate a forward feedback cycle, resulting in the release of pro-inflammatory microparticles (MPs), Hb, heme, and iron, and the breakdown products generated by hemolysis have remarkable effects on immunological functions (**Figure 3**).

### 3.1 Healthy RBCs and immune regulation

RBCs modulate innate and adaptive immunity mainly through their surface molecules (proteins, lipids, and carbohydrates) and potent antioxidant capacity (102); they express large amounts of the key complement regulators, CD55, CD59, and complement receptor type 1 (CR1, also referred to as CD35), where CD55 inactivates C3 convertases generated by all three complement activation pathways, CD59 prevents membrane attack complex formation by preventing C9 incorporation, and CR1 recognizes collagen-like regions of C1q, mannose-binding lectin, C3b, and C4b, to remove complement-tagged inflammatory particles. For example, in patients with HIV, the virus binds to RBCs via C1q-CR1 interaction (103). Further, CR1 is decreased on RBCs in patients with coronavirus disease 2019 (COVID-19), resulting in consistent inflammatory responses and tissue damage (104, 105).

Toll-like receptor 9 (TLR9), a nucleic acid sensing receptor, is expressed on the surface of mammalian RBCs. Under basal conditions, RBCs bind cell-free mitochondrial DNA (mtDNA) through TLR9 and mediate DNA scavenging to prevent unnecessary inflammation (106). Further, in the context of inflammation, TLR9 binds to CpG-containing DNA derived from bacteria, plasmodia, and mitochondria, which drives innate immune activation and red cell clearance (107). Erythroid-specific TLR9 deletion blocks erythrophagocytosis and decreases local and systemic cytokine production (107). During viral pneumonia and sepsis secondary to COVID-19, RBCs also exhibit protein oxidation, together with decreased antioxidant capacity, increased glycolysis, an altered membrane lipidome, and elevated mtDNA binding, which may contribute to anemia and disease severity (102, 108).

Moreover, RBCs can induce DCs toward an immature/tolerogenic phenotype in response to lipopolysaccharide (LPS), through a CD47-dependent mechanism (109). Mechanosensing by RBCs also ensures exposure of splenic type-2 conventional DCs to blood flow, allowing them to capture circulating antigens, while retaining them in the spleen through CD55-CD97 signaling (110); the same mechanism is also important for marginal B cell retention and function (111). In addition, Duffy blood antigen, primarily expressed on the surface of RBCs, can potently bind to multiple chemokines (112, 113).

Transfusion of fresh RBCs under noninflammatory conditions will reduce RBC clearance and therefore lessen macrophage loading with heme, as well as up-regulating heme oxygenase (HO), shifting macrophages toward the anti-inflammatory M2 state (112). Protein factors released from RBCs, such as Hb and peroxiredoxin II, can sustain normal and leukemic T cell growth and survival (113). RBCs can also synergize with TCR/CD3-mediated activation signals and enhance T cell survival and proliferation through a calcineurin-dependent mechanism (111).

### 3.2 Mito^+^ RBCs and immune regulation

Programmed mitochondrial removal occurs during normal erythropoiesis (114). A hypoxia-inducible factor-mediated metabolic switch and consequent activation of the ubiquitin-proteasome system precede, and are necessary for, autophagic mitochondria removal, and disruption of this pathway leads to accumulation of RBCs carrying mitochondria (Mito^+^ RBCs) (115). This process is defective in patients with systemic lupus erythematosus (SLE) (115) and sickle cell disease (SCD) (116), as well as in aged mtDNA mutator mice (117). In patients with SLE, Mito^+^ RBC levels are correlated with disease activity, and antibody-mediated Mito^+^ RBC internalization by macrophages induces type I interferon (IFN-I) production through cGAS/STING activation (115), while Mito^+^ RBCs may contribute to SCD pathophysiology via high reactive oxygen species (ROS) production (118).

### 3.3 Oxidized or senescent RBCs and immune regulation

RBCs are frequently exposed to various stressful conditions during their lifespan, including oxidative stress, osmotic shock, and mechanical squeezing (119), and consequently accumulate damage which influences their functions. Senescent RBCs show pathologic properties, including decreased deformability (120), MP release (121), increased hemin-carrying Hb (122), and surface antigen modification (123). RBC senescence occurs alongside oxidative stress and in turn becomes a source of ROS, which serves as an important signal of RBC senescence (124). Accumulation of oxidized lipids, such as 4-hydroxynonenal, may induce vascular inflammation (125, 126). At the molecular level, the major features of senescent RBCs are Band 3 clustering or breakdown (127), PS externalization (128), loss of glycophorin A, and reduction of CD47 expression (124). Consequently, senescent RBCs lose the ability to control LPS-induced DC maturation (129).

Oxidized or senescent RBCs or RBC-derived MPs are potential modifiers of T cell responses, which enhance mitogen-driven T cell proliferation and apoptosis through an antigen presenting cell- and cell contact-dependent mechanism, and regulate IFN-γ production from T helper 1 cells (124). Moreover, oxidized RBCs release Hb, heme, and iron which are both sources of radicals and able to activate ECs (130) and innate immune cells, such as monocytes (131), in a proinflammatory manner, as detailed below (see section 3.4). Stored RBCs display senescence-related changes, such as reduced structural integrity, MP release, and iron overload, and the transfusion of stored RBCs exacerbates existing lung inflammation and promotes lung injury, due to loss of Duffy antigen expression and their chemokine scavenging function during storage (132). Further, rapid clearance of transfused stored RBCs by macrophages polarizes the macrophages toward the classical M1 phenotype, with a huge Hb iron load (112, 133). In addition, packed RBCs suppress T cell proliferation via cell-cell contact and inhibit T cell activation via ROS-dependent signaling (134).

### 3.4 Hemolysis and immune regulation

RBCs are highly differentiated cells with an elegant structure that allows them to survive under continuous shear stress when transiting, making them ideal messengers between distant organs. The erythrocyte membrane skeleton is a polygonal 2D lattice structure, consisting of lipids, proteins, and carbohydrates (135, 136). The skeleton attaches to the cell membrane through the spectrin-actin junctional complex (adducin, dematin, and P4.1 interact with band 3, GPC/D, and Glut1) and the ankyrin complex (137). Disorders of the RBC cytoskeleton or dehydration cause hemolytic anemia, which is associated with altered immune regulation, as hemolysis breakdown products, including hemoglobin, heme, and iron, have remarkable effects on immunological functions (138).

#### 3.4.1 Hemoglobin

Hemoglobin (Hb) is an iron-containing protein in RBCs formed from globin and heme (Fe^2+^ protoporphyrin-IX). When large amounts of Hb are released into the plasma from damaged RBCs, the scavenger protein haptoglobin (Hp) can rapidly bind with cell-free Hb, to generate a Hb-Hp complex, which neutralizes the pro-oxidative effects of Hb (139). When Hp binding capacity is saturated, heme in free Hb is easily oxidized to hemin (Fe^3+^ protoporphyrin-IX) in the circulation. Free Hb triggers vascular and organ dysfunction through extravascular translocation, NO inactivation, oxidative reactions, hemin release, and activation of downstream signaling pathways (see section 3.4.2) (139). Hp-Hb complexes bind to the CD163 receptor expressed on macrophages and hepatocytes and are subsequently digested, releasing heme into the cytoplasm (140). Free Hb enhances platelet activation by binding to ADP, as well as by abrogating the inhibitory effect of NO (141).

#### 3.4.2 Heme

Heme is an important iron-containing porphyrin molecule and with crucial roles in cell protection, apoptosis, inflammation, and immune disorders (142). Hydrophobic hemin intercalates into cell membranes. Hydrogen peroxide from various sources splits the heme ring and releases free redox-active iron, which catalytically amplifies ROS production. Consequently, heme regulates inflammation mainly by acting as a pro-oxidant in macrophages, neutrophils, and ECs (143). Furthermore, heme can selectively bind to receptors, transcription factors, and enzymes (89).

Heme stimulates monocyte differentiation into splenic red pulp macrophages and bone marrow macrophages by promoting degradation of the transcriptional repressor, BACH1, and consequent expression of the transcription factor, SPI-C (144). Heme can also act as a pro-inflammatory second hit in macrophages by aggravating LPS-induced TLR4 signaling, or induce an anti-inflammatory response (M2 macrophages) via induction of SPI-C and HO-1, an inducible isoform of HO (145). Moreover, heme impairs phagocytosis by inhibiting cytoskeleton dynamics through the DOCT8/Cdc42 signaling pathway (146). Heme can also induce Treg expansion in purified T cell-monocyte cocultures by upregulating HO-1 in nonclassical monocytes (138).

Heme promotes neutrophil migration by stimulating macrophage-derived leukotriene B4 (147) and activating protein kinase C and G-protein-coupled receptors in neutrophils (148, 149), which induce chemokine expression and ROS production. During neutrophil development in patients with SCD, heme regulates neutrophil differentiation and can cause defective oxidative burst through HO-1 induction (150). Heme can also inhibit neutrophil apoptosis *in vitro* through the phosphoinositide 3-kinase (PI3K) and NF-κB pathways (151). Further, heme can induce neutrophil extracellular trap (NET) formation through ROS signaling, to protect the host against infections (152); however, in patients with SCD, NETs can enhance the adherence of erythrocytes and platelets to the endothelium and induce vascular occlusion or lung injury (153).

Free heme interacts with ECs and stimulates the expression of adhesion molecules, including intercellular adhesion molecule 1 (ICAM-1), endothelial cell adhesion molecule (ECAM), vascular cell adhesion molecule 1 (VCAM-1), P-selectin, and others, through heme-mediated ROS and NF-κB signaling pathways (154). Leukocytes attach tightly to endothelium through adhesion molecules and migrate to tissue parenchyma, which promotes vascular occlusion and subsequent tissue ischemia (154, 155). In addition, cell-free heme and heme-loaded microvesicles activate the complement system via the alternative pathway in both serum and on the surface of ECs. Heme also upregulates P selectin, C3aR, and C5aR expression, and downregulates that of CD46, on ECs, which contributes to endothelial damage and vascular occlusion in patients with SCD (156).

#### 3.4.3 Iron

Free heme is catabolized by HO into three products: biliverdin, carbon monoxide (CO), and Fe^2+^ (142), where biliverdin is converted to bilirubin, and both CO and bilirubin have potent anti-inflammatory and antioxidant properties, whereas Fe^2+^ enhances oxidative stress, thereby promoting ferroptosis (157). Fe^2+^ binds to the iron storage protein, ferritin, which has cytoprotective and anti-oxidative effects, as well as a role in iron storage. Ferritin was first discovered as a suppressor of granulocyte and macrophage production in 1981 (158). Further studies demonstrated that ferritin comprises two functionally distinct subunits: ferritin H and L (159). H-ferritin can suppress T cell proliferation in response to mitogens and impairs B cell maturation (159), as well as helping to mediate the protective effect of HO-1 against oxidative stress (160). Moreover, H-ferritin is a negative regulator of CXC chemokine receptor 4 in receptor-mediated cell migration (161). L-ferritin overexpression in LPS-induced Raw264.7 cells can significantly decrease the production of pro-inflammatory cytokines (TNF-α, IL-1β) and NO and inhibit MAPK and NF-κB activation (162).

## 4 Immunomodulatory effects of CECs

The term CECs refers to immature erythroid cells, including erythroblasts and reticulocytes, which are physiologically enriched in the spleen and cord blood of neonates, but rare in adult bone marrow (45). CECs are characterized by expression of CD71 and glycoprotein A (CD235a)/glycoprotein A-related protein (Ter119), as CD71^+^TER119^+^ cells in mice and CD71^+^CD235a^+^ cells in humans. CD71 is also known as transferrin receptor 1 (TfR-1), a type II transmembrane protein important in cellular iron uptake and iron metabolism (163). CD71 is a surface marker for erythroid cells from BFU-E to reticulocytes, which first appears in BFU-E, reaches its highest expression levels in Baso-E and Poly-E cells, then declines in Ortho-E cells, and finally disappears in mature erythrocytes (33).

**Figure 4:**
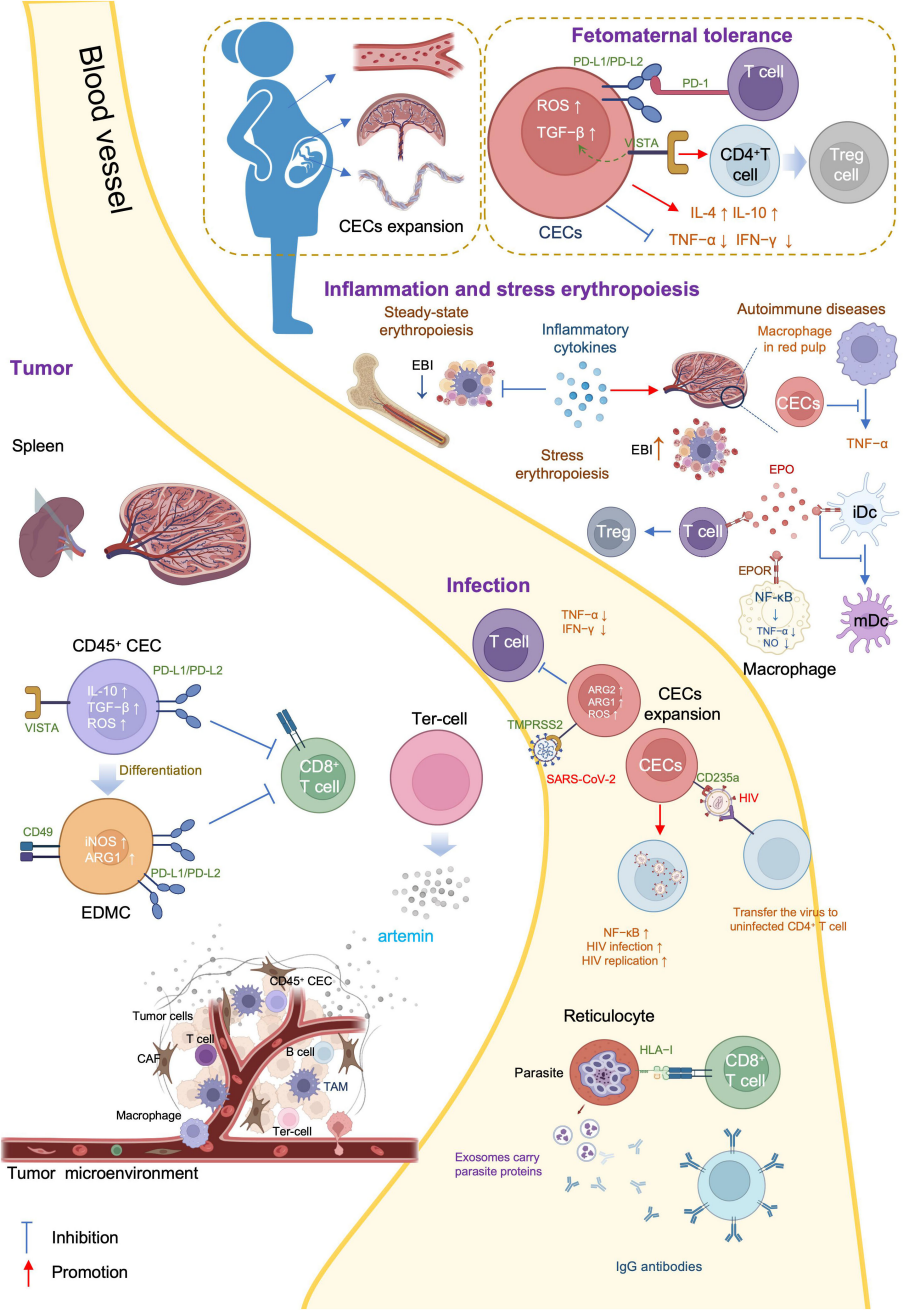
Mechanisms and immunoregulation effects of CECs.

There are three general CEC subtypes: early-stage CECs, EDMCs, and late-state CECs, each with differing immunosuppressive abilities. Erythropoietic tracking showed that CD45^+^CD71^+^TER119^+^ cells are enriched with stage I–III precursors, while CD45^-^CD71^+^TER119^+^ cells contain more terminally differentiated stage III–V erythroid cells (164). Recent studies have indicated that CECs at the earliest stages are more potent immune response suppressors (164–166). The various surface markers and functional properties of the three CEC subtypes are summarized in **Table 3**, the immunomodulatory effects of the CECs are summarized in **Table 4** and **Figure 4**, and the immunomodulatory effects of the CECs in diseases are summarized in **Table 5**.

**Table 3:** Phenotypes of the CECs.

	early-stage CECs	EDMCs	late-state CECs
Markers: mouse	CD45^+^CD71^+^TER119^+^	CD45^-^CD71^+^TER119^+^ CD11b^+^Gr1^+^	CD45^-^CD71^+^TER119^+^
Markers: human	CD45^+^CD71^+^CD235a^+^	CD45^-^CD71^+^CD235a^+^ CD11b^+^CD33^+^HLA-DR^-^	CD45^-^CD71^+^CD235a^+^
Immunosuppressive ability	high	high	low
Key mediators of suppression	ROS IL-10 TGF-β ARG1 ARG2 VISTA PD-1/PD-L1	iNOS ARG1 PD-L1 PD-L2 CD49	Artemin

**Table 4:** Mechanisms of the CECs in immunoregulation.

Mechanism		Diseases	Effects
Arginase	ARG1	COVID-19 patients and anemia	Suppress cytokines produced by T cells Suppress T cells proliferation
	ARG2	COVID-19 patients and anemia Neonates and pregnancy	Suppress cytokines produced by T cells Suppress T cells proliferation Suppress cytokine produced by myeloid cells
ROS		COVID-19 patients, anemia and tumor HIV patients Neonates and pregnancy	Suppress cytokines produced by T cells Suppress T cells proliferation Promote HIV replication and trans- infection in T cells Suppress cytokine produced by myeloid cells
Cytokines	TGF-β	Tumor Neonates and pregnancy	Suppress cytokines produced by T cells Suppress T cells proliferation Promote Tregs differentiation
	IL-10	Tumor	Suppress T cells proliferation
	Artemin	Tumor	Promote tumor growth
	IL-22	Anemia	Decrease EPCs number in bone marrow
Immune checkpoints	PD-1/PD-L1	Tumor Neonates and pregnancy	Suppress cytokines produced by T cells Suppress T cells proliferation Suppress cytokines produced by T cells
	VISTA	Tumor Neonates and pregnancy	Suppress cytokines produced by T cells Suppress T cells proliferation Promote Tregs differentiation

**Table 5:** Immunomodulatory effects of the CECs in diseases.

	Disease or animal model	Mechanisms	Effects	Ref.
Neonatal	*Listeria monocytogenes* infection and Escherichia coli infection	ROS and ARG2	CECs in PBMCs suppress the production of TNF-α by monocytes and IFN-γ by T cells *in vitro*	(167, 168)
	*Bordetella pertussis* infection	ARG2	CECs in neonatal mouse spleen inhibit the immune response against *B. pertussis* infection *in vivo*, and CD71^+^CD235a^+^ cells in human cord blood inhibit T and B cell function *in vitro*	(169, 170)
	VISTA KO mice	VISTA, TGF-β, inhibition of the Akt signaling pathway	Splenic CECs secret TGF-β to promote CD4^+^ T cell differentiation into Tregs	(171)
	RRV infection	Not mentioned	Reduce TNF-α production	(172)
	Endotoxin challenge and polymicrobial sepsis	Not mentioned	CECs do not modify murine mortality	(173)
Pregnancy	CECs depletion in pregnant mice	ARG2 and PD-1	Maternal CD71^+^ erythroid cells inhibit allogeneic response to promote fetomaternal tolerance	(174, 175)
	Pregnant mice	TGF-β-dependent mechanisms	TGF-β facilitates the differentiation of CD34^+^ HSPCs into CECs without impacting HSPCs proliferation	(55)
	Allogeneic mouse model and IBD patients	VISTA, TGF-β, and ROS	CEC number decreased and CECs express lower levels of inhibitory molecules including VISTA, TGF-β and ROS	(176)
Infection	HIV patients	ROS	CECs enhances NF-κB in CD4^+^ T cells to facilitate HIV infection. CECs bind to HIV-1 via CD235a and subsequently transfer the virus to uninfected CD4^+^ T cells	(177)
	Nosocomial infections and sepsis	Not mentioned	Low levels of RBCs and high levels of IL-6 and IFN-γ may contribute to the expansion of CECs	(178)
	COVID-19	ARG2, ARG1, and ROS	CECs suppress TNF-α and IFN-γ secretion by CD4^+^ and CD8^+^T cells *in vitro*	(179)
	*P. vivax* infection	Cytotoxic CD8^+^ T cells	Reticulocytes highly express HLA-I	(180)
	*P. yoelii* infection	Exosomes regulation	Reticulocytes in BALB/c mice secrete exosomes carry parasite proteins and are involved in immune modulation.	(181)
	*Salmonella* infection	Myd88/TRIF	Erythropoiesis occurs in the spleen	(182)
Inflammation	T cell-induced colitis mouse	phagocytosis-associated pathway	CECs suppress TNF-α expression in red pulp macrophages *in vitro*	(183)
	SoJIA patients	ARG2?	Suppress the secretion of IL-1β, IL-6, and IL-8 by monocytes	(184)
	Chronic inflammation in Turpentine-induced sterile abscess	Not mentioned	Inflammation impacts the late stages of mammalian erythroid development	(185)
	Zymosan-induced generalized inflammation	Heme-dependent activation of SPI-C	Inflammatory signals induce stress erythropoiesis to maintain erythroid homeostasis	(79)
Tumor	LLC patients and B16-F10 mouse	ROS	CD45^+^ EPC accumulate in the spleen and impair CD8^+^ T cell function	(164)
	HCC patients (CD45^+^ CECs)	IL-10, TGF-β, and ROS	Suppress T cells production and proliferation through the NF-κB, STAT-3, TGF-β, and ROS pathways in a paracrine and cell-cell contact manner	(165)
	HCC patients and tumor-bearing mice (CD45^-^ CECs)	Artemin	Splenic Ter-Cells secret neurotrophic factor artemin in the blood and promotes tumor progression by inducing Caspase-9 Thr125 phosphorylation and upregulating TRIOBP and ITGB5 expression	(186)
	Virus-associated solid tumors patients and mouse with melanoma	ROS, PD-L1, PD-L2, VISTA, and TGF-β	CD45^+^CECs express more ROS, PD-L1/PD-L2, and VISTA to suppress T cell function through TGF-β	(187)
	Patients with advanced tumors and B16-F10 lung metastasis mouse model	PD-L1, PD-L2, iNOS, ARG1, and CD49	EDMCs inhibit CD8^+^ T cell proliferation and IFN-γ production, and reduce the anti-PD-1/PD-L1 treatment efficacy	(166)
Anemia	EPOR-HM mice	EPO	CD45^+^ CECs expansion	(188)
	COVID-19 patients	Lysis and phagocytosis	Infected/damaged CECs are eliminated by lysis or/and phagocytosis.	(179)
	Anemia patients without proliferative diseases and NHA mice	ARG and ROS	In mice, CD45^+^CECs express high levels of ARG2 and ROS, CECs expansion-induced L-arginine depletion suppresses T-cell responses in the spleen. In humans, CECs expand and express ARG1 and ARG2 that suppress IFN-γ production by T cells	(189)
	LLC patients and B16-F10 mouse	ROS	CD45^+^ CECs are robust ROS producers and suppressors of TCR-stimulated CD8^+^ T cell proliferation	(164)
	*P. falciparum* or *P. vivax* infected patients	Uninfected reticulocytes retention in the spleen	Uninfected reticulocytes congestion in the red-pulp	(190)
	*P. chabaudi* AS infected mice	Insufficient erythropoiesis	EPO-induced proliferation of early EPOR^+^ erythroid progenitors is suppressed	(191)
	MDS and CKD patients	IL-22	BM erythroid precursor cell frequency and number decrease	(192)

### 4.1 CECs in neonatal and pregnancy

Erythroid cells play a crucial role in immunological regulation during the neonatal period and in maternal-fetal tolerance. Mouse placental erythroid cells are mainly CD45^+^ and secrete the chemokines, CCL2, CCL3, CCL4, and CXCL1 (193). Further, CECs are abundant in mouse neonatal spleen and human cord blood, and possess unique immunosuppressive properties (167). CECs are abundant in the liver of children with biliary atresia (BA), and suppress the activation of hepatic mononuclear cells (172). Further, CECs are numerous in the peripheral blood of human newborns, but decline rapidly by 4 weeks of age (168).

CECs influence neonatal infections through various mechanisms. *Bordetella pertussis* is a common neonatal respiratory tract pathogen and CECs prevent the recruitment of immune cells to the mucosal infection site (167). CECs from human newborn peripheral blood mononuclear cells (PBMCs) suppress TNF-α production by CD14^+^ monocytes and IFN-γ production by T cells (168). Further, ablation of CECs enhances the innate immune response by increasing the production of protective cytokines, including IL-17, IFN-γ, TNF-α, and IL-12 in *B. pertussis*-infected lungs (169, 170).

L-arginine is essential for T cell proliferation and function (194). Arginase (ARG) depletes L-arginine, thereby inhibiting T cells, and is encoded by two recently-discovered genes, *Arg1* and *Arg2* (195). ARG1 is expressed in the cytosol, whereas ARG2 localizes to mitochondria. Neonatal and human cord blood CECs express ARG2 and ablation of CECs augments *B. pertussis*-specific T cell responses in the lung and spleen on re-infection or vaccination (170). In addition, ablation of CECs also induces enhanced systemic and mucosal *B. pertussis*-specific antibody responses (170). Accordingly, CECs in human cord blood can suppress T and B cell functions *in vitro* (170). Regarding innate immunity, CECs inhibit *B. pertussis* phagocytosis via ARG2 *in vitro* (169, 170). Such effects of CECs facilitate intestinal colonization with commensal microbes during the neonatal period (167). Depletion of CECs in neonatal mice renders them more resistant to infections by *Listeria monocytogenes*, *Escherichia coli*, and *B. pertussis*, indicating the protective effects against neonatal infectious diseases (167, 168); however, ablation of CD71^+^ cells failed to modify neonatal mortality in either a model of endotoxin challenge or a model of polymicrobial sepsis (173).

BA is a rare and progressive disease that develops in early infancy (196). Rhesus rotavirus (RRV) infection of neonatal mice induces an obstructive cholangiopathy, which is similar to BA (197). CECs expand in the liver of children with BA or RRV-infected mice and suppress the immune response by reducing TNF-α production. Preemptive depletion of hepatic CD71^+^ erythroid cells in neonatal mice augments the number of effector lymphocytes and delays RRV infection of the liver and extrahepatic bile duct, suppressing bile duct injury (172). Clearance of CECs before RRV infection renders mice resistant to RRV-induced BA, while repopulation of CD71^+^ erythroid cells after RRV inoculation promotes long-term survival (172).

CEC-mediated immunosuppression is crucial for fetomaternal tolerance. Both BALB/c and C57BL/6 female mice, and human women are enriched for CECs (198). Further, analysis of 155 umbilical cord blood samples showed that the proportion of CECs was 50-fold higher in cord blood than that in maternal blood (199). Erythropoiesis becomes active during pregnancy, and erythrocytes significantly expand in the peripheral blood (200). TGF-β has an important role in regulating the erythroid lineage differentiation potential of HSCs (201, 202). CECs in pregnant mice express more PD-L1/PD-L2 and suppress T cells expressing programmed cell death protein-1 (PD-1) at the fetomaternal interface (174). Maternal CECs inhibit IFN-γ and TNF-α production to protect the fetus against the allogeneic response. Further, fetal liver CECs also exhibit immunosuppressive activity. A recent transcriptional study demonstrated expression of high levels of galectin-9, galectin-1, and VISTA on the surface of neonatal splenic CECs. CD71^+^VISTA^+^ cells produce more TGF-β than CD71^+^VISTA^−^ cells, and can promote CD4^+^ T cell differentiation into Tregs (171); however, CECs in human cord blood express negligible amounts of VISTA. Indeed, VISTA expression levels are significantly higher in placental CECs than those in cord blood (171). Thus, both maternal and fetal CECs are essential for fetomaternal tolerance (175). Accordingly, depletion of CECs in pregnant mice induces a proinflammatory immune response, by reducing IL-4 and IL-10 production, while increasing TNF-α and IL-6 levels in placental tissues, which in turn results in fetal resorption (175, 203); however, in pregnant women with inflammatory bowel disease (IBD), CECs are decreased in the peripheral blood, cord blood, and placenta tissue, and express lower levels of inhibitory molecules, including VISTA, TGF-β, and ROS. Accordingly, pregnant women with IBD have lower levels of Tregs and increased immune-activation. Patients with IBD are more likely to have a pro-inflammatory environment in the gastrointestinal tract, which leads to impairment of CECs during pregnancy (176).

### 4.2 CECs in infection

CECs not only function during neonatal infections, they participate in various infections throughout life.

Acquired immune deficiency syndrome is a systemic disease caused by human immunodeficiency virus (HIV), the genome of which comprises two copies of a 9749 nucleotide sequence packaged in each virion (204). CECs are expanded in the peripheral blood of patients with HIV and there is a positive correlation between CEC frequency and plasma viral load. When cocultured with CD4^+^ T cells, CECs exacerbate HIV-1 infection/replication, by enhancing NF-κB activation in CD4^+^ T cells to facilitate HIV infection (177). Meanwhile, CECs bind to HIV-1 via CD235a and subsequently transfer the virus to uninfected CD4^+^ T cells. Moreover, in the presence of antiretroviral therapy, CECs from HIV-infected individuals contain infective viral particles, which mediate HIV-1 trans-infection of CD4^+^ T cells (177). CECs are also significantly expanded and possess immunosuppressive properties in the blood of patients with COVID-19. With high levels of ARG2, ARG1, and ROS, CECs mediate immunosuppression by inhibiting CD4^+^ and CD8^+^ T cell production of TNF-α and IFN-γ *in vitro* (179). Furthermore, CD45^+^ CECs express ACE2, TMPRSS2, CD147, and CD26 and can be infected with SARS-CoV-2 (179).

CECs are also expanded in adult patients with sepsis and serve as predictors of 30-day mortality as well as nosocomial infection development. Low levels of RBCs and high levels of IL-6 and IFN-γ may contribute to the expansion of CECs in sepsis (178). During *Salmonella* infection, accumulation of CECs in the spleen and increased EPO production are dependent on Myd88/TRIF signaling (182); EPO neutralization reduces the population of CECs in the spleen and slightly improves the host immune response (182).

Malaria is an insect-borne infection caused by the bite of *Anopheles* mosquitoes, and a major global health problem, with approximately 247 million cases worldwide in 2021 and many more residents of endemic areas having asymptomatic parasitemia (chronic malaria) (205). Different species of malaria parasites exhibit distinct tropism (206). *Plasmodium falciparum* can invade all stages of erythrocytes while *Plasmodium vivax* and *Plasmodium cynomolgi* invade only reticulocytes (207, 208). *P. vivax* is the most widely distributed human malaria parasite and exhibits a strong preference for immature reticulocytes, with CD71 acting as an anchor receptor (209, 210). Reticulocytes have a more complex and enriched metabolic profile than mature erythrocytes, providing metabolic reservoirs for malaria parasites (206). *P. vivax*-infected reticulocytes express high levels of human leukocyte antigen class I (HLA-I), which can be specifically detected by cytotoxic CD8^+^ T cells to protect against intracellular parasites (180). In BALB/c mice, reticulocytes can secrete exosomes when infected by the reticulocyte-tropic non-lethal *Plasmodium yoelii* 17X strain (211). These reticulocyte-derived exosomes carry parasite proteins and are involved in antigen presentation. Mice immunized using purified exosomes produce IgG antibodies that can recognize *P. yoelii*-infected RBCs and show increased survival time and altered reticulocyte cell tropism of the parasite (181). Furthermore, during *P. vivax* infection, parasites invariably affect bone marrow CD71^+^ cells, inducing dyserythropoiesis and ineffective erythropoiesis (212). Identification and characterization of the reticulocyte receptor, metabolism, and the underlying mechanisms involved in malaria may provide insights to inform the development of novel antimalarial drugs and vaccines.

### 4.3 CECs in inflammation

Inflammation is the automatic defense response to tissue injury, and can be classified as acute and chronic, according to its duration. Inflammation modifies bone marrow hematopoiesis towards innate immune effector cells at the expense of lymphoid cells and erythrocytes (79). Inflammatory cytokines, such as TNF-α, limit steady-state erythropoiesis and promote granulopoiesis. Further, mature granulocytes contact the central macrophage of EBIs and alter EBI structures, leading to increased numbers of maturing granulocytes and fewer erythroid precursors (213). In chronic inflammation resulting from sterile abscesses, erythropoiesis is impaired at Ter119^+^ stages of erythroid development (185). Although inflammation inhibits erythropoiesis in the bone marrow, inflammatory signals induce stress erythropoiesis in the spleen, to maintain erythroid homeostasis. Inflammatory signaling through TLRs enhances erythrophagocytosis by splenic macrophages and augments expression of the transcription factor, SPI-C. In turn, SPI-C couples with TLR signaling to promote the expression of *Gdf15* and *Bmp4*, which encode ligands that initiate the expansion of stress erythroid progenitors in the spleen (79). The spleen is the largest secondary lymphoid organ, and has a wide range of immunologic functions alongside its roles in erythropoiesis, and splenic erythropoiesis alters the histological structure of spleen to become rich in granulomatous lesions and devoid of clear separation between red and white pulp (214).

Autoimmune diseases comprise a range of disorders in which the immune response to self-antigens results in tissue damage or dysfunction (215). In patients with autoimmune diseases, CECs can inhibit inflammatory responses to prevent excessive inflammation and injury. Experimental autoimmune encephalomyelitis (EAE) is an autoimmune disease mainly mediated by specific sensitized CD4^+^ T cells, which serves as the best experimental model reflecting the autoimmune pathogenesis of human multiple sclerosis (216), and iron-deficient mice fail to develop EAE (217). Management using EPO or its non-erythropoietic derivatives consistently decreases EAE-associated TNF-α, IL-1β, and IL-1Ra production in the spinal cord, and IFN-γ by peripheral lymphocytes, which ameliorates chronic murine EAE (218). IBD inflammation spreads systemically and can cause complications, such as arthritis, cachexia, and anemia. In a CD45-deficient Rag1-deficient mouse model of T cell-induced colitis, an increased number of erythroid progenitors are found in the spleen. These CECs can suppress TNF-α expression in red pulp macrophages in a phagocytosis-dependent manner (183). Further, erythropoiesis-related genes are upregulated in PBMCs of patients with systemic-onset juvenile idiopathic arthritis (SoJIA) (184), while active SoJIA-driven CECs co-cultured with healthy donor monocytes suppress IL-1β, IL-6, and IL-8 secretion. Although ARG2 is the top upregulated gene in SoJIA-driven CECs, cytokine production from monocytes remains suppressed when they are treated using an arginase inhibitor (184).

### 4.4 CECs in tumor

Tumors are complex ecosystems, comprising tumor cells and various non-neoplastic cells (219), where non-neoplastic cells in the tumor microenvironment play critical roles in cancer development. Targeting the tumor microenvironment is considered a promising approach for cancer intervention (220). CECs are abundant in both the tumor microenvironment and the circulation and their levels can be used to predict tumor recurrence (165).

Tumor-associated myeloid cells include myeloid-derived suppressor cells (MDSCs), tumor-associated macrophages (TAMs), and neutrophils (221), which are important immune cell populations in the tumor microenvironment that are crucial for immune checkpoint blockade efficacy (222). MDSCs can be divided into at least two major subsets: mononuclear MDSCs (M-MDSCs, CD11b^+^Ly6G^-^Ly6C^high^) and polymorphonuclear MDSCs (PMN-MDSCs, CD11b^+^Ly6G^+^Ly6C^low^) (223), where M-MDSCs exert more robust immunosuppression than PMN-MDSCs. Further, erythroid cells can differentiate into myeloid cells in tumors and mediate immunosuppression. Lineage tracking in patients with cancer and tumor-bearing mice revealed that > 30% of erythroid progenitor cells lose their developmental potential and switch to the myeloid lineage, and that these erythroid differentiated myeloid cells (EDMCs) are similar to their myeloid-originated counterparts at the transcription level (166). The phenotypes of EDMCs are CD45^+^CD235a^+^CD71^+^CD11b^+^CD33^+^HLA-DR^-^ in patients with cancer and CD45^+^Ter119^+^CD71^+^CD11b^+^Gr1^+^ in tumor-bearing mice. EDMCs express more immune inhibitory molecules, including PD-L1, PD-L2, iNOS, ARG1, and CD49, than MDSCs, which may endow EDMCs with the ability to inhibit CD8^+^ T cell proliferation and IFN-γ production. Accordingly, EDMCs reduce the efficacy of anti-PD-1/PD-L1 treatment (166).

In tumors, CD45^+^ CECs exert a strong immune suppressive function, mainly by regulating T cells. In Lewis lung cancer, CD45^+^ CECs are induced by tumor growth-associated extramedullary hematopoiesis (EMH) in the spleen and their transcriptome closely resembles that of MDSCs. As robust immunosuppressors, CD45^+^ CECs hinder both CD8^+^ T cell priming in the spleen and effector function in peripheral tissues (164). In hepatocellular carcinoma (HCC) tissues, CD45^+^ CEC numbers are higher than those of CD45^-^ CECs. Further, CD45^+^ CECs from patients with HCC inhibit CD4^+^ T cell proliferation and differentiation and suppress CD8^+^ T cell proliferation and cytotoxicity by generating factors including ROS, IL-10, and TGF-β (165). In patients with virus-associated solid tumors, substantially greater expansion of CECs occurs in the blood compared with that in healthy controls. CD45^+^ CECs have more immunosuppressive properties than their CD45^-^ counterparts, mediated by higher levels of ROS, PD-L1/PD-L2, and VISTA. Further, the abundance of CECs in the circulation may be associated with anemia (187). Moreover, CECs in mice with melanoma secrete artemin, while this is not the case for VISTA^+^ CECs in patients with virus-associated solid tumors (187).

CD45^-^ CECs have lower immunosuppression abilities than their CD45^+^ counterparts; however, they also play a crucial role in promoting tumor progression. One population of tumor-induced erythroblast-like cells (CD45^-^Ter119^+^CD71^+^, Ter-cells) derived from MEPs (186, 224), accumulate in the spleen of patients with terminal cancer and secret artemin, where artemin is a neurotrophic factor with an important role in cancer progression through its induction of Caspase-9 Thr125 phosphorylation, to maintain cell survival, and upregulation of TRIOBP and ITGB5 expression, to promote invasion. Blocking artemin, or its receptor, GFRα3, signaling inhibits HCC growth *in vivo* (186). In this context, the phenotype of Ter-cells is CD45^-^Ter119^+^ CD71^+^CD41^+^CD44^+^, and they mainly exist in the spleen of advanced-tumor bearing hosts; however, a few can also be found in the tumor. TGF-β and Smad3 activation contribute to Ter-cell generation. Moreover, serum artemin levels in patients with HCC are correlated with poor prognosis (186).

### 4.5 CECs in anemia

Anemia is a common blood disorder characterized by a decreased number of RBCs in the peripheral blood, which is defined as a hemoglobin level less than the 5th percentile for age (225). Anemia is the main cause of EPC expansion by increasing EPO concentration in response to oxygen deficit (226). In mice with anemia, CD45^+^ CECs expand in the spleen and express high levels of ARG2 and ROS. CEC expansion-induced L-arginine depletion suppresses T cell responses. In patients with anemia, CECs expand in the peripheral blood and express ARG1 and ARG2, which suppress IFN-γ production by T cells (189). Furthermore, human erythroleukemia-derived erythroid cell lines, including K562, HEL92.1.7, and TF-1, which express multiple erythroid-lineage markers, such as CD71 and CD235a, suppress T cells in an ARG- and ROS-dependent manner (189). Serum levels of IL-22 are increased in patients with anemia secondary to chronic kidney disease and myelodysplastic syndromes, and the IL-22 receptor, IL-22RA1, is present on erythroid precursors, with blockade of IL-22 signaling alleviating anemia in mice (192).

Anemia is also a common feature of sepsis (227). In patients with sepsis, RBC levels are negatively associated with CD45^+^ CEC frequency, suggesting that anemia may lead to CEC expansion through the EPO pathway (178). EPO can induce the expansion of CD45^+^ CECs, while EPO neutralization prevents infection-related CEC accumulation (188). In patients with COVID19, SARS-CoV-2 infection is associated with lower blood oxygen levels and the numbers of CECs in the blood are negatively correlated with hemoglobin levels; this may be due to the elimination of infected/damaged CECs by lysis and/or phagocytosis. Dexamethasone enhances the maturation of CECs to RBCs by downregulating ACE2 and TMPRSS in a dose-dependent manner (179).

In addition, anemia is very common among patients with cancer and tumor bearing animal models; approximately 30%–90% of patients with cancer have varying degrees of anemia, depending on the type of cancer (228). Immunosuppressive CECs can be detected in patients with cancer and anemia. Further, hematocrit, HGB levels, and mature RBC counts are decreased in the blood of mice after prolonged tumor-bearing, and HGB is negatively correlated with numbers of splenic CECs. Tumor-initiated anemia and EMH may act synergistically to cause splenic CEC accumulation (164): anemia induces EMH, whereas terminal differentiation is blocked in the presence of tumors. RNA sequencing of CD45^+^ and CD45^-^ CECs generated by anemia induced in different ways revealed that CD45^+^ CECs differ significantly from their CD45^-^ counterparts, particularly regarding signature genes defining the erythrocyte lineage and immunosuppression. Notably, ROS and NOX-2 are highly expressed in CD45^+^ CECs, particularly those from tumor-bearing individuals (164). EPO has been widely used to overcome hypoxia in patients with cancer. Recombinant human EPO and erythropoiesis-stimulating agents can promote EPC differentiation and maturation to RBCs, and thereby effectively treat anemia; however, these agents do not prolong the survival of patients with cancer (229–231). Immune checkpoint inhibitors (ICIs) targeting co-inhibitory molecules, including PD-1, PD-L1, and cytotoxic T lymphocyte-associated antigen 4 (CTLA-4), have been widely applied in the therapy of various tumors (232); however, EPO treatment in patients receiving anti-PD-L1 therapy reduce the therapeutic effects of this monoclonal antibody (187); the underlying mechanism involves EPO induction of continual differentiation of CECs into EDMCs, which mediate systemic immunosuppression against immune surveillance (166).

## 5 Future applications

### 5.1 Manipulation of CECs

**Figure 5:**
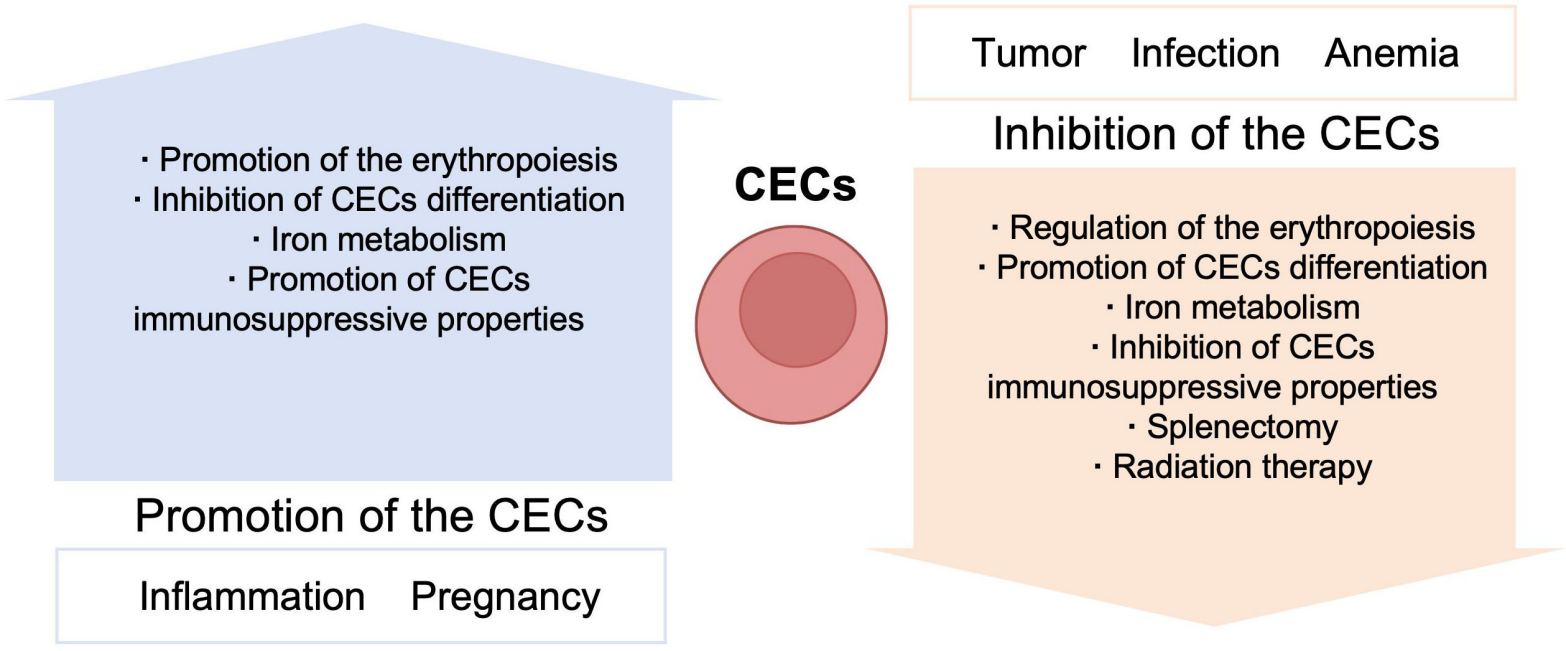
Manipulation of the CECs.

Erythroid cells participate in several immune conditions and have important roles in regulating immune responses. Further, CECs may have beneficial effects in fetomaternal tolerance and autoimmune diseases; however, in contexts including infection, tumor, and anemia, CECs appear to exert detrimental effects (**Table 6**). Thus, further understanding of the immune regulatory mechanisms used by erythroid cells can provide new insights into pathogenic mechanisms, and CECs may serve as a novel target in immunological therapies (**Figure 5**). CECs can have opposite effects in different diseases, and different measures could be selected to manipulate CECs, according to context; for example, promotion/inhibition of CEC expansion, inhibition/promotion of CEC differentiation, and inhibition/promotion of CEC immunosuppressive properties.

**Table 6:** Immunomodulatory effects of the CECs.

	Clinical scenario	CECs	Effect	Outcome
Neonatal	infection	promoted	Immunosuppression	Detrimental
	physiological	abundant	Immunosuppression and fetomaternal tolerance	Beneficial
Pregnancy	IBD	decreased	Impaired immunosuppressive functions	Beneficial
	physiological	promoted	Enhance the erythropoiesis and fetomaternal tolerance	Beneficial
Infection	virus	promoted	Immunosuppression and facilitate to infection	Detrimental
	bacteria	promoted	Immunosuppression	Detrimental
	malaria	host	Exosomes from infected reticulocytes modulate immune response	—
Inflammation	inflammation	↓↑	Impaired immunosuppressive functions	Beneficial
	Autoimmune diseases	decreased	Impaired immunosuppressive functions	Beneficial
Tumor	CD45^+^CECs	promoted	Immunosuppression	Detrimental
	CD45^-^CECs	promoted	Artemin secretion to promote tumor growth	Detrimental
Anemia		promoted	Immunosuppression	Detrimental

“↓↑”: Bone marrow erythropoiesis decreased and spleen erythropoiesis promoted.

Under normal conditions, CECs suppress the immune response to protect tissue from immunologic injury; however, CECs may be impaired in autoimmune diseases and chronic inflammation. Thus, promoting CEC expansion may be beneficial in inflammatory disorders. In contrast, in patients with tumors, infection, or anemia, CECs expand and arrest at an early stage, to inhibit immune activity; therefore, preventing CEC expansion and promoting CEC differentiation may be promising therapies to attenuate immune evasion and enhance immune responses in these contexts.

#### 5.1.1 Targeting CEC expansion signals

EPO is crucial in regulating the late stages of erythropoiesis, and EPO and EPO derivatives are widely used to treat different types of anemia (233–235). Clinical studies have demonstrated that EPO can significantly improve the management of anemia in patients with chronic renal insufficiency (236). Further, EPO administration for patients with cancer significantly improves hematological responses and decreases the need for RBC transfusion (237); however, caution is required in patients with cancer and anemia as, while EPO may influence the curative effect of ICI therapy by restoring Ter-cell numbers and serum artemin concentration (166), EPOR are present on various types of tumor cell and tumor cell lines, and EPO/EPOR may contribute to tumor progression and metastatic progression (238). EPO can also strongly suppress immune system activation and protect injured tissues from apoptosis, suggesting that it may be a promising therapeutic target in autoimmune diseases, allergy, and organ transplantation. As EPOR is expressed on various immune and tumor cells, the interactions between EPO and these cell types requires further study.

Other factors involved in stress erythropoiesis can also be targeted to modulate CEC expansion. GCs are established immunosuppressive steroid molecules secreted by the adrenal gland and regulated by the hypothalamic-pituitary-adrenal axis, and are widely used in the treatment of various immune disorders (239, 240). GCs can also be used to treat anemia by modeling human stress erythropoiesis, as they can both induce monocyte differentiation to EBI macrophages (241) and directly target EBI macrophages to promote erythroid expansion (242). Further, GCs can aid in the treatment of EPO-resistant anemia by stimulating progenitor self-renewal (243), while, in healthy humans, GC injection also accelerates erythropoiesis and increases total hemoglobin mass, which may help to prevent altitude sickness (244). Although GC application in immune disorders has been widely studied, whether GCs can be used to modulate CECs in these diseases awaits further in-depth investigations.

#### 5.1.2 Promoting CEC differentiation

CECs expand and arrest in the early stages of maturation in patients with cancer and suppress immune responses. Therefore, promoting CEC differentiation is a novel therapeutic strategy to diminish the tumor-promoting effects of CECs.

TGF-β triggers differentiation arrest and promotes CEC expansion, as well as functioning as the main effector cytokine of CECs that regulate immunosuppression (245). Further, TGF-β and downstream Smad3 activation are important in splenic Ter-cell generation (186). Inhibitors targeting TGF-β and Smad2/3 signaling can stimulate CEC differentiation and promote their maturation, thereby neutralizing their suppressive effects (202, 246), and have been proven effective in mouse models of cancer. In addition, TGF-β-promoted immune escape of carcinoma cells can be flexibly treated using ionizing radiation combined with hyperthermia and ICIs. Numerous anti-cancer pharmacological interventions targeting TGF-β have undergone pre-clinical and clinical stage studies; however, although several anti-TGF-β-based immunotherapies were effective in preclinical trails (247–249), the results of subsequent clinical trials were disappointing, due to low efficacy and safety issues (250). Thus, further research to explore optimal combinations with other chemotherapies and improve specificity is needed.

p38 MAPK signaling is important in regulating erythropoiesis, and restrains EPC differentiation by regulating active GATA-1 degradation (251). Further, p38 MAPK signaling contributes to several biological functions, including inflammation and tumorigenesis, as well as cell proliferation, differentiation, apoptosis, and senescence (252–254). Therefore, p38 signaling inhibitors may be beneficial in patients with cancer as they have anti-tumor effects and can promote CEC maturation; however, similar to TGF-β inhibitors, although some p38 MAPK inhibitors have completed phase I and II trials, the results of clinical trials have been unsatisfactory due to high levels of systemic toxicity (254). Thus, further research is warranted to facilitate more comprehensive understanding of p38 MAPK signaling.

mTOR belongs to the PI3K-related kinase family of serine/threonine protein kinases and acts with Forkhead-box-class-O3 (FoxO3) to regulate erythropoiesis (255). FoxO3 inhibits mTOR and promotes CEC differentiation by inducing cell cycle exit of early-stage EPCs during ineffective erythropoiesis (255). mTOR inhibitors or FoxO3 inducers may be used to reduce ineffective erythropoiesis by promoting CEC maturation and inhibiting CEC proliferation (256); however, first-generation mTOR inhibitors showed limited sensitivity (257). Additional research is needed to enhance this type of therapy and overcome resistance.

Caspases are negative regulators of erythropoiesis through caspase-mediated degradation of the transcription factor, GATA-1 (258). In chronic inflammation, inflammasomes activate caspase-1 and skew the differentiation of HSPCs toward myeloid cells, resulting in neutrophilia and anemia. Caspase-1 inhibition rapidly upregulates GATA1 and promotes HSPC differentiation into erythroid cells (259). Caspase-1 is also involved in inflammatory processes and autoinflammation; therefore, it is of great interest to evaluate the effects of caspase inhibitors, such as colchicine (260), VRT-18858, VRT-043198, and sulfasalazine (261), on CEC expansion and differentiation in inflammation-associated anemia and autoimmune diseases.

Iron, a necessary component of hemoglobin and myoglobin, is essential in hemoglobin synthesis and erythroid cell proliferation (60), and iron deficiency leads to anemia. Thus, targeting iron and its metabolism is an effective way to ameliorate ineffective erythropoiesis and reduce accumulation of early-stage CECs. Several new agents to modulate iron metabolism, such as anti-hepcidin antibody (LY2787106) (262), anti-ferroportin antibody (LY2928057) (263), and anti-matriptase-2 antibody (RAP-536L and RLYB331), have been investigated (264), and are all beneficial in the treatment of anemia; hence, drug combinations incorporating these agents represent a potential superior option. RLYB331 prevents iron overload, ameliorates ineffective erythropoiesis, and limits the formation of toxic α-chain, while RAP-536L efficiently corrects anemia in β-thalassemic model mice. Combination treatment with RLYB331 and RAP-536L integrates their advantages, including hepcidin upregulation, alleviation of iron overload, and amelioration of ineffective erythropoiesis (264). Moreover, inflammation-inducible cytokines can directly suppress CEC differentiation as well as blocking intestinal iron absorption and causing iron-restricted erythropoiesis (265). Thus, therapies targeting pro-inflammatory cytokines can also promote CEC differentiation by increasing iron availability.

#### 5.1.3 Modulation of CEC immunosuppressive properties

CECs modulate immune responses through multiple mechanisms, including L-arginine depletion by ARG, ROS, cytokines, and immune checkpoints. ARG inhibitors, such as boronic acid derivatives or L-arginine supplementation, may diminish the inhibitory effects of CECs on immune responses (189, 266). Similar to ARG inhibitors, targeting of ROS-generating proteins, including NOX enzymes, or use of a ROS inhibitor, such as N-acetylcysteine, may be helpful therapeutic strategies for autoimmune conditions or cancer (267). Further, targeting cytokines secreted by CECs is a promising strategy to attenuate CEC-induced immune evasion. TGF-β superfamily inhibitors can both ameliorate CEC suppressive effects and cooperate with EPO to promote RBC production and alleviate anemia (268); neutralization of TGF-β also reduces CEC expansion. Thus, targeting TGF-β signaling is a potentially promising strategy; however, as discussed above, a number of challenges are yet to be overcome. Late-stage CECs secrete artemin to promote tumor growth and invasiveness, and anti-artemin neutralizing antibody can inhibit tumor growth and increase the survival of tumor-bearing mice. Thus, the clinical utility of targeting artemin or related signaling pathways warrants exploration. Targeting immune checkpoints has revolutionized clinical oncology and antibodies targeting the PD-1/PD-L1 axis have proven effective in cancer therapy. CECs mediate immune response suppression by the PD-L1/PD-1 axis, and ICIs may, at least partially, suppress the tumor-promoting effects of CECs.

Importantly, mechanisms by which CECs induce immunosuppression also overlap with those used by many other immunomodulatory cells, including Tregs, MDSCs, and TAMs, among others. Therefore, therapeutic strategies targeting these mechanisms to modulate the properties of CECs may also influence other immunomodulatory cells, leading to unexpected effects.

#### 5.1.4 Splenectomy and radiation

Splenomegaly occurs in patients with anemia or advanced cancer, where the spleen becomes a central organ of EMH, which generates suppressive cells, including CECs and myeloid cells (269). Splenectomy is associated with longer hospital stay and longer time to chemotherapy in patients with cancer but has no impact on overall or disease-free survival (270); however, for patients with advanced epithelial ovarian cancer or with splenic involvement, spleen resection is associated with longer survival (271, 272). Moreover, splenectomy leads to the depletion of MDSCs and promotes the activation of anti-tumor immunity (269), hence it may be beneficial for some patients with advanced cancer. Although there have been several clinical studies of splenectomy, more preclinical and clinical investigations are required.

Radiation is often used as an adjuvant therapy for tumors, and exhibits substantial versatility and efficacy in cancer treatment (273). Local irradiation can significantly decrease tumor-induced Ter-cell accumulation in the murine spleen (224). Further, patients with cancer who received local tumor ionizing radiation (IR) alongside PD-1 therapy exhibited IR-mediated reduction of Ter-cells, artemin, and GFRα3 (an artemin signaling partner associated with tumor regression) (224). Hence, radiation is an effective cancer treatment, and understanding the interactions when immunotherapies are combined with radiotherapy warrants further study.

### 5.2 Future applications of RBCs

Targeting RBCs is an underdeveloped therapeutic strategy; however, with their strong ability to contribute to material exchange and high immunocompatibility, RBCs have potential as drug delivery carriers (274, 275). RBC membrane-coated polymeric nanoparticles can effectively deliver doxorubicin in a mouse model of lymphoma (275). Further, RBC extracellular vesicles (RBCEVs) are taken up by leukemia cells with high efficiency, and may serve as a valid vehicle to deliver antisense oligonucleotides to leukemia cells (274). RBCEVs are also used as a vehicle for osteoclast-targeted delivery of anti-miR-214 oligonucleotides. TBP-CP05 is a functional peptide which binds to both CD63 on RBCEVs and receptors on osteoclasts, and TBP-CP05 binds with RBCEVs through CP05 and endows them with osteoclast-targeting ability. Intravenous injection of osteoclast-targeting RBCEVs significantly inhibits osteoclast activity, elevates osteoblast activity, and improves bone density in osteoporotic mice (276). Hence, RBCs have huge potential in cancer and clinical therapy as a novel type of nanoparticle-based RNA drug vehicle.

RBCs also have potential applications in disease diagnosis, prognosis, and monitoring (277). A considerable fraction of cell-free mtDNA, which is associated with trauma, autoimmune disease, sepsis, malignancy, cellular injury, and organ dysfunction, binds to the outer surface of RBCs and can serve as a biomarker (278–280). Furthermore, long DNA fragments which cover most nuclear and mitochondrial genome regions can be detected in RBCs from patients with cancer (281).

## 6 Perspectives on erythroid cell research

Erythroid cells possess complex immunoregulation functions at different stages of their development. Overall, the available evidence demonstrates the broad range of immunological properties possessed by these most abundant, but less appreciated, cells. Further studies should clarify the roles of erythroid cells at different stages of development and in various diseases and their underlying mechanisms, which could inform the development of new therapeutic strategies. Furthermore, recent studies have revealed erythropoiesis in the skull and dura (282, 283), and tissue-specific functions in these contexts are also of interest.
